# 

**Published:** 1895-01-26

**Authors:** 


**Notices of Births, Deaths, and Marriages** are inserted in this column at a charge of 2s. 6d. for an announcement not exceeding four lines.

**Notice to Advertisers and Subscribers.**
All Advertisements, Orders for Copies of The Hospital, and Business Communications should be addressed to **The Manager, NOT TO THE EDITOR,** The Hospital, 428, Strand, London, W.C.
The Cost of Subscription, post free, is as follows:—Per week, 2½d.; per quarter, 2s. 8d.; per half-year, 5s. 3d.; per annum, 10s. 6d. Foreign:—Per Annum, 15s. 2d.; payable, in all cases, in advance.

### NOTICE TO CORRESPONDENTS.

All MS., Letters, Books for Review, and other matters intended for the Editor should be addressed The Editor, The Lodge, Porchester Square, London, W.
The Editor cannot undertake to return rejected MS., even when accompanied by stamped directed envelope.

**"Burdett's Hospital Annual," 1895,**
In Active Preparation.
Sixth year of publication. About 900 pp. Scarlet cloth, gilt lettered. Price 5s. A most complete guide to British, Colonial, Indian, and American Hospitals, Dispensaries, Nursing and Convalescent Institutions, and Asylums. A few copies of the "Annual" for 1894 may still be obtained on application to the Publishers, The Scientific Press (Limited), 428, Strand, London, W.C.

**NOTICE.—The Index for Vol. XVI. (ending September 24th, 1894) is on sale at the Office of this Journal, Price 2½d., post free.**

### Scraps and Gleanings.

The Queen has sent a present of game for the use of patients in St. George's Hospital.

The Duke of York has sent a present of game to the Royal Eye Hospital, Southwark.

The Goldsmiths' Company has given £100 to the North Eastern Hospital for Children, Shoreditch.

We learn that the Sultan of Turkey is having an asylum for incurables of all creeds erected not far from his palace.

Dr. Barnardo's annual supper for waifs and strays took place last week, when 2,000 children took part in the entertainment.

Mr. Brunner, M.P. for Northwich, has sent a cheque of £500 to the National Society for the Prevention of Cruelty to Children.

One of the new knights, Sir John Baker, M.P., has offered £500 to the Royal Portsmouth Hospital towards a convalescent home for that institution.

Mr. Edward Courage, of Shenfield, near Brentwood, has made the munificent donation of £1,100 to the Brentwood and District Cottage Hospital.

The Duke and Duchess of York will visit Sheffield in May to open a new block of buildings which have been added to the public hospital and dispensary and which are to cost £50,000.

The condition of drainage of the Taunton Hospital being in a highly unsatisfactory condition, an outlay of £100 will be involved in placing the building in sanitary order.

Mr. Leopold de Rothschild will preside at the annual festival dinner on April 23rd next, at the Whitehall Rooms, in aid of the Metropolitan Hospital, Kingsland Road.

### THE STUDENT OF MEDICINE.

### APPOINTMENTS.

W. Beatty, M.D., appointed Physician to the Adelaide Hospital, Dublin. H. T. Bewley, M.D., appointed Assistant Physician to the Adelaide Hospital, Dublin. R. P. Crosbie, M.B., B.Ch., B.A.O.R.U.I., appointed Assistant House Surgeon to the South Charitable Infirmary and County Hospital, Cork. W. A. Day, M.R.C.S.Eng., L.R.C.P.Edin., appointed Medical Officer for the Kirkleatham District of Guisborough Union. S. De Butts, M.D., appointed Anæsthetist to the Royal Ear Hospital, Frith Street, Soho. H. Fairfax, L.S.A., appointed Medical Officer for No. 13 District of the West Ham Union. Dr. Fisher, appointed Assistant Port Medical Officer for the Milton and Conyer Districts. G. R. Fortune, L.R.C.P., L.R.C.S.Edin., appointed Police Surgeon for the Central Division of Glasgow. J. K. Goodall, L.R.C.P., L.R.C.S.Edin., appointed Medical Officer of Health for Brimington. H. Head, M.D., appointed Consulting Physician to the Adelaide Hospital, Dublin. W. Jameson, L.R.C.P., L.R.C.S. Edin., appointed Medical Officer of Health for Ashover. B. J. Lee, L.S.A., reappointed District Medical Officer to the Chesterfield District Council. W. W. Leigh, L.R.C.P.Edin., M.R.C.S. Eng., appointed Medical Officer for the Llanfabon District of the Merthyr Union. J. Little, M.D., appointed Senior Physician to the Adelaide Hospital, Dublin, *vice* H. Head, M.D. R. McLay, M.B.Glasg., appointed Medical Officer for the Parish of Tarbolton. R. Martin, L.R.C.P. and S.Edin., L.F.P.S.Glasg., appointed Medical Attendant Royal Irish Constabulary, at Gilford. J. C. Matheson, M.B., C.M., appointed House Surgeon to the Royal Ear Hospital, Frith Street, Soho. G. Parker, M.A.Camb., M.D., M.R.C.S.Eng., appointed Honorary Physician to the Bristol Dispensary. Mr. J. Parsons, appointed Medical Officer for the Fourth District of the Alton Union. J. Reid, M.B., B.Ch., B.A.O.R.U.I., appointed House Surgeon to the South Charitable Infirmary and County Hospital, Cork. W. J. Richard, M.A., M.B., C.M.Glasg., appointed Medical Officer, Govan Poorhouse and Asylum, Merryflatts, Govan. W. H. Richards, L.S.A., appointed Medical Officer and Public Vaccinator for the St. Mellon's and Rumney Parishes of the Newport Union. W. G. Richardson, M.B., B.S., F.R.C.S., appointed Surgical Registrar to the Royal Infirmary, Newcastle-on-Tyne. J. A. Scott, M.D., appointed Pathologist and Bacteriologist to the Adelaide Hospital, Dublin. W. Shears, M.B.Lond., M.R.C.S., L.R.C.P., appointed Junior House Surgeon to the Scarborough Hospital and Dispensary. J. A. M. Thomson, L.R.C.P., L.R.C.S.I., appointed Medical Officer of Health to the Bradford-on-Avon Rural District Council. A. H. Tubby, M.S.Lond., F.R.C.S.Eng., appointed an Assistant Surgeon to the Westminster Hospital. Mr. C. W. Vine appointed Medical Officer for the No. 4 District of the Portsea Island Union.

### VACANCIES.

The following vacancies are announced this week, the amount of salary in each case being given after the name of the institution:

### Non-Resident Medical Officers.

Hospital for Diseases of the Throat, Golden Square, W.—Registrar and Pathologist. Appointment for six months, which may be renewed. Honorarium of 25 guineas a year is attached to the appointment. Applications to the Secretary by February 1st.

University of Edinburgh.—Additional Examiner in Natural History and Clinical Surgery. Period of office four years. Salary £75 per annum in each case, with £10 per annum for travelling and other expenses in the case of an additional examiner not resident in Edinburgh or the immediate neighbourhood. An additional allowance is made to the Examiner in Natural History for examining for Graduates in Arts. Applications to L. J. Grant, Interim Secretary, University Court, University of Edinburgh, by February 6th.

### THE STUDENT OF MEDICINE—*continued.*

**Resident Medical Officers.**

Central London Ophthalmic Hospital, 238A, Gray's Inn Road, W.C.—House Surgeon. Applications to the Secretary by February 12th.

Wirral Children's Hospital, Woodchurch Road, Birkenhead.—House Surgeon. Salary £50 a year, with board, lodging on the premises, and washing. Applications to P. W. Atkin, Honorary Secretary, 25, Lord Street, Liverpool, by January 30th.

North-Eastern Hospital for Children, Hackney Road, Shoreditch, N.E.—Junior House Physician; doubly qualified. Appointment for six months. No salary, but board and lodging (including washing) provided. Applications to the Secretary, 27, Clement's Lane, E.C., by February 13th.

Parish of Ronsay and Egilshay.—Resident Medical Officer. Salary £51 per annum. Applications to the Inspector of the Poor, Ronsay, by January 31st.

### Medical Appointments.

NORTH-EASTERN HOSPITAL for CHILDREN.
HACKNEY ROAD, SHOREDITCH, N.E.

